# Gestational weight gain and optimal wellness (GLOW): rationale and methods for a randomized controlled trial of a lifestyle intervention among pregnant women with overweight or obesity

**DOI:** 10.1186/s12884-019-2293-8

**Published:** 2019-04-30

**Authors:** Susan D. Brown, Monique M. Hedderson, Samantha F. Ehrlich, Maren N. Galarce, Ai-Lin Tsai, Charles P. Quesenberry, Assiamira Ferrara

**Affiliations:** 1 Division of Research, Kaiser Permanente Northern California, Oakland, CA 94612 USA; 2 The University of Tennessee, Knoxville, TN USA

**Keywords:** Pregnancy, Obesity, Gestational weight gain, Lifestyle intervention, Clinical trial, Protocol

## Abstract

**Background:**

Excess gestational weight gain (GWG) is common among women with overweight or obesity, increasing their risks for pregnancy complications, delivering a large infant, and postpartum weight retention. To date, only intensive interventions have had success and few interventions have been designed for implementation in healthcare settings.

**Methods:**

We describe the development, rationale, and methods of GLOW (GestationaL Weight Gain and Optimal Wellness), a randomized controlled trial evaluating the efficacy of a lifestyle intervention to prevent excess GWG among racially/ethnically diverse women with overweight or obesity in an integrated healthcare delivery system. Participants in Kaiser Permanente Northern California will be randomized, within 2 weeks of completing a study baseline clinic visit at 10 weeks’ gestation, to either usual medical care or a multi-component pregnancy lifestyle intervention adapted from the Diabetes Prevention Program (target *N* = 400). Informed by focus groups with patients and designed to be feasible in a clinical setting, the intervention will include 13 weekly individual sessions (11 delivered by telephone) focused on behavior change for weight management, healthy eating, physical activity, and stress management. Outcomes will be assessed in women and their infants from randomization to 12 months postpartum. The primary outcome is GWG. Secondary outcomes include changes in diet and physical activity during pregnancy and infant birthweight. Exploratory outcomes include cardiometabolic profile assessed via pregnancy blood samples and cord blood samples; and postpartum weight retention and infant anthropometrics up to 12 months of age. The trial includes systematic approaches to enhance intervention fidelity, intervention adherence, and participant retention in trial assessments.

**Discussion:**

GLOW is among few trials targeting excess GWG among diverse women with overweight or obesity in a healthcare setting, with long-term maternal and infant outcomes assessed up to 12 months after delivery. This evaluation of a multi-component intervention is designed to produce generalizable results to inform potential adoption of the intervention in clinical settings.

**Trial registration:**

ClinicalTrials.gov (NCT02130232): submitted April 30, 2014; posted May 5, 2014.

**Electronic supplementary material:**

The online version of this article (10.1186/s12884-019-2293-8) contains supplementary material, which is available to authorized users.

## Background

Exceeding the Institute of Medicine (IOM) guidelines for gestational weight gain has significant implications for women and their infants [1]. Up to 64% of women with overweight or obesity experience excess gestational weight gain (GWG) [2, 3], which elevates their risks for gestational diabetes mellitus, Cesarean delivery, postpartum weight retention, and obesity in later years [4–7], and for having an infant who is large-for-gestational age [8] and at increased risk for overweight or obesity in childhood [9].

Until recently, GWG interventions in women with overweight or obesity have had limited success [10] and few trials included diverse samples, despite excess GWG significantly impacting women across racial and ethnic groups [11]. Trials arising from the Lifestyle Interventions for Expectant Moms (LIFE-Moms) consortium now show that intensive behavioral lifestyle interventions, such as in-person counseling or in-person counseling paired with partial meal replacements, prevent excess GWG among women with overweight or obesity [12]. Still, intensive interventions may not be feasible for many women and may be difficult to implement in healthcare delivery settings. In addition, data are needed on long-term maternal and infant health outcomes beyond the immediate postpartum period [13].

Here we provide an overview of the GestationaL Weight Gain and Optimal Wellness (GLOW) randomized controlled trial. The trial compares usual medical care to a mainly telephone-based lifestyle intervention designed to be feasible in healthcare settings, which we hypothesize will prevent excess GWG among pregnant women with overweight or obesity. The primary outcome is rate of GWG per week, measured as a continuous variable and whether it meets the 2009 IOM guidelines [1]. Secondary GWG outcomes include weekly rate of GWG between study clinic visit assessments at 10 weeks’ and 32 weeks’ gestation, total GWG, and whether total GWG meets the IOM guidelines. Other secondary outcomes include changes in diet and physical activity during pregnancy and infant birthweight. Exploratory outcomes include changes in cardiometabolic markers assessed at 10 weeks’ and 32 weeks’ gestation, cardiometabolic profile in cord blood, postpartum weight retention at 6 and 12 months, and children’s anthropometrics at up to 12 months of age.

## Methods

**Fig. 1:**
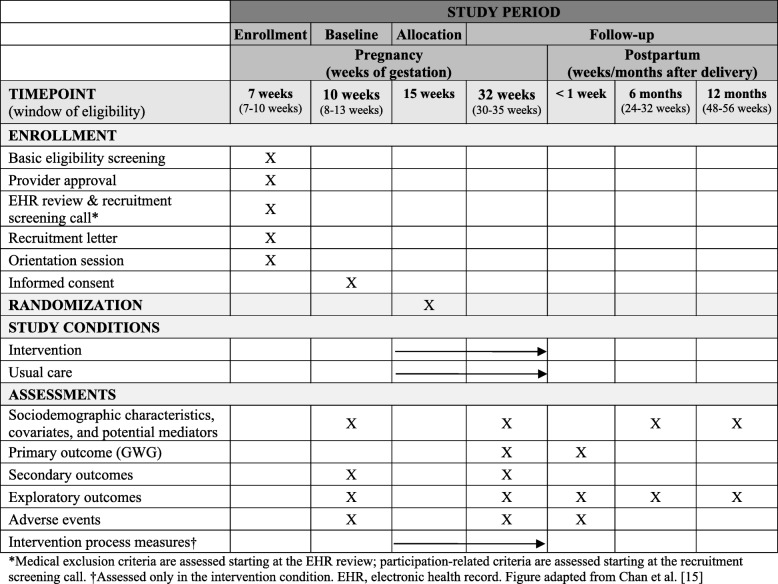
Sequence of participant enrollment, intervention, and assessment activities: The GLOW trial

GLOW is a two-arm, parallel group randomized controlled trial testing the efficacy of a multi-component pregnancy lifestyle intervention adapted from the Diabetes Prevention Program (DPP) [14] and delivered primarily via telemedicine, as compared to usual medical care. GLOW is funded by the National Institutes of Health (R01 HD073572; PI: Ferrara); registered at ClinicalTrials.gov (NCT02130232; submitted April 30, 2014, posted May 5, 2014); and approved by the Kaiser Foundation Research Institute Human Subjects Committee. This article follows the SPIRIT (Standard Protocol Items: Recommendations for Interventional Trials) guidelines for reporting clinical trial protocols [15]. The sequence of trial activities for participant enrollment, intervention, and assessment appears in Fig. 1.

### Setting

GLOW will be conducted within Kaiser Permanente Northern California (KPNC), a large pre-paid, integrated healthcare delivery system serving over 4 million members broadly representative of the population in the underlying geographic area [16]. GLOW will recruit pregnant women from four medical centers, in Oakland, San Leandro, Santa Clara/Fremont, and Walnut Creek. These medical centers were selected based on their racial/ethnic diversity and high volume of deliveries.

### Eligibility and recruitment

GLOW aims to recruit a racially and ethnically diverse group of 400 participants to increase generalizability. Pregnant women receiving care at the selected medical centers will be first identified in the electronic health record (EHR) system. Only those < 8 weeks’ gestational age will be evaluated to confirm eligibility criteria: ≥18 years of age, pre-pregnancy body mass index (BMI) between 25 and 40 kg/m^2^ (based on weight measured in the clinical setting within 6 months before the last menstrual period), and a singleton pregnancy. Eligibility will be further assessed through a tiered process beginning with approval from medical providers to contact each patient, and EHR review. Medical exclusion criteria that may impact dietary and/or outcome assessment will be determined through the EHR review and a recruitment screening call. These include being pregnant with multiples; pregnancy loss; fertility-assisted pregnancy; diagnosed with diabetes before pregnancy; current gestational diabetes (although those diagnosed with gestational diabetes after randomization remain in the study); current uncontrolled hypertension; thyroid disease diagnosed in last 30 days; history of cardiovascular disease, cancer, lung disease or serious gastrointestinal disease; history of eating disorder or bariatric surgery; serious mental illness; recent history of mood or anxiety disorder; and drug or alcohol use disorder. Exclusion criteria that may interfere with full participation in the trial will be assessed starting at the recruitment call. These include extensive travel plans during pregnancy; plans to move out of the area or to become pregnant in the year after delivery; not having reliable transportation or telephone access; inability to communicate in English; disinterest in being randomized; and currently being over 13 weeks of gestation.

At the recruitment call, women will be asked to verify their pre-pregnancy weight as assessed in the EHR, since this measured weight will be used to calculate the GWG outcomes for all women in the trial; and for GWG goal setting for those randomized to the intervention condition. Women with a discrepancy of > 5 lbs. between the pre-pregnancy weight assessed in the EHR and by self-report will not be considered eligible to participate in the baseline clinic visit.

### Consent and randomization

Trained research assistants will obtain informed consent at the baseline clinic visit at 10 weeks’ gestation. Following this visit, women will complete at least two of three 24-h diet recalls by telephone within one week. Women will also wear an accelerometer for at least 10 h a day over at least four out of seven days (described below in section 2.6.). Women who complete fewer than two diet recalls or provide fewer than four 10-h days of accelerometer data will not be eligible for randomization. Women will also no longer be eligible for randomization if they experience a pregnancy loss between the baseline clinic visit and the time of randomization. Women with a pregnancy loss after randomization will be withdrawn from the study.

Eligible women who consent and complete the baseline assessment will be randomly assigned to either the usual care or intervention conditions. To generate each participant’s random assignment, unblinded study staff will use MinimPy, a free open-source computerized randomization program [17]. The adaptive randomization procedure is designed to ensure that equal numbers of patients are assigned to each study condition and that the two conditions remain balanced overall, within each medical center, and on each level of key characteristics: age (< 30 and ≥ 30 years), pre-gravid BMI (< 30, 30–34.9, and ≥ 35 kg/m^2^), and race/ethnicity (Asian/Pacific Islander; Black; Latina; White; and multiethnic/other/unknown). The process cannot be manipulated by study personnel. The assignment will be recorded in a Microsoft Access tracking database, where it is only accessible to non-blinded personnel (e.g., lifestyle coaches). The research assistants conducting data collection, study investigators, biostatistician, and programmer analyst will be blinded to condition assignment.

Steps taken to ensure the confidentiality of information about prospective and enrolled participants will include keeping all study information in a secured computer system used only for research, and identifying participants by a unique study identification number rather than name or medical record number. No information from the study will be placed in participants’ medical records or shared with the Kaiser Foundation Health Plan.

### Usual care

Women randomized to the usual care condition will receive standard KPNC prenatal medical care. This includes an initial prenatal visit at 7–10 weeks’ gestation; an additional 7 routine prenatal visits between 16 weeks’ gestation and delivery; and periodic health education newsletters, which include the IOM GWG guidelines and advice on healthy eating and activity in pregnancy. Medical staff routinely weigh patients at each prenatal visit.

### Lifestyle intervention

In addition to usual care as described above, women randomized to the intervention condition will receive a multi-component pregnancy lifestyle intervention called “Getting in Balance.” The Getting in Balance intervention was adapted from the DPP [14] and our prior research [18, 19] to be feasible among pregnant women for possible adoption in a healthcare system setting. The intervention targets behavior changes for weight management, healthy eating, physical activity, and stress management to meet a trial goal of gaining at the lower limit of the IOM-recommended range for GWG: 7 kg for women with overweight (BMI 25.0–29.9 kg/m^2^) and 5 kg for women with obesity (BMI ≥ 30.0 kg/m^2^).

#### Theoretical basis and behavior change techniques

The intervention follows a step-wise, phased approach to behavior change based on Bandura’s social cognitive theory [20, 21] and the Transtheoretical model [22]. Coaches will use motivational interviewing techniques to support participant autonomy and behavior change, e.g., by guiding participants to identify personal pros and cons of lifestyle changes [23, 24]. Behavior change techniques, categorized according to a standard taxonomy [25], include *self-monitoring of* and *feedback on behavior* (e.g., daily self-monitoring of dietary and physical activity behaviors); *self-monitoring of* and *feedback on outcomes of behavior* (e.g., daily self-monitoring of weight); *goal setting*; *review of behavior goals* (e.g., reviewing dietary self-monitoring records and modifying goals accordingly); *problem solving*; *action planning*; *social support*; *prompts and cues* (e.g., encouraging participants to create visual cues to be active); *self-rewards* (e.g., encouraging participants to give themselves non-food rewards after achieving goals); *providing information about health consequences* (e.g., information on the benefits of healthy eating during pregnancy); and providing information from a *credible source* (e.g., a lifestyle coach).

#### Patient input

To refine the intervention to be feasible among pregnant women, we explored patients’ perspectives on weight gain, lifestyle behaviors, and proposed intervention materials using a mixed methods approach. In 2013, we recruited pregnant women with overweight or obesity from two of the proposed KPNC medical facilities, Oakland and Santa Clara. After obtaining informed consent, trained moderators administered a brief survey followed by a 90-min focus group discussion, for which participants were reimbursed with a $50 gift card. The sample included 25 participants, 36% (*n* = 9) with overweight and 64% (*n* = 16) with obesity, from diverse backgrounds: 36% Black (*n* = 9), 32% White (*n* = 8), 24% Latina (*n* = 6), 4% Asian (*n* = 1), and 4% multiracial (*n* = 1). Sixteen percent had a high school education or less (*n* = 4), 32% some college education or a 2-year degree (*n* = 8), and 52% a four-year college degree (*n* = 13). Survey data indicated frequent discrepancies between the IOM guidelines and women’s beliefs about how much they should gain during their current pregnancy: 48% of women (*n* = 12) reported an ideal weight gain amount that would exceed the IOM guidelines for their BMI category, 48% (*n* = 12) reported an amount within the guidelines, and 4% (*n* = 1) reported an amount below the guidelines (overall mean [SD] for ideal weight gain amount, 10.4 [3.2] kg; median 11.4 kg; range 6.8–18.1 kg). Using an item adapted from prior research [26, 27], only 4% (*n* = 1) reported that they weigh themselves at least daily; equal proportions reported that they weigh themselves at least weekly, monthly, or not at all (32% [*n* = 8] in each category). In focus group discussions, women described both facilitators of healthy weight gain (e.g., partners offering healthy foods) and barriers such as partners offering unhealthy foods, fatigue, nausea, stress, depression, and long work hours. Women also described having made changes to the way they managed their weight in the current pregnancy, which ranged from increasing regulation (e.g., reading food labels more often; packing lunches as a healthier option than eating out) to decreasing regulation (e.g., considering pregnancy as a “freedom period”; feeling tired and thus “[putting] less stress on myself” to eat healthy). Some also described aversions to looking at their weight and the need for a weight gain chart to be accompanied by non-judgmental professional support.

Patient input reinforced including key elements in the intervention, such as health information about the IOM guidelines and the rationale for weight gain goals. With many women estimating their appropriate GWG as above the guidelines, we deemed it important to counsel women on the rationale for gaining less weight than they may otherwise have assumed. Additional key elements included the rationale for self-weighing, and ways to use a scale and weight gain chart effectively; social support; stress management; and pregnancy-specific barriers to weight management and lifestyle behavior change.

#### Intervention structure and content

The intervention will be delivered by trained lifestyle coaches through 13 individual weekly counseling sessions: a first in-person session (60 min in duration), followed by 11 telephone sessions (20 min each) and a final in-person session (60 min; Table 1). Coaches will be registered dietitians trained in physical activity, weight management during pregnancy, motivational interviewing, and behavior change techniques. Coaches will receive regular group and individual supervision from master’s- and doctoral-level study staff.

**Table 1:** Overview of core sessions for the *Getting in Balance* intervention: The GLOW trial

Session title	Topic	Modality (duration)
1. Welcome to *Getting in Balance*	▪ Introduction to intervention goals ▪ Demonstration and instruction on how to self-monitor weight and dietary intake	In-person (60 min)
2. Getting Started with Healthy Eating	▪ Diet quality; portion sizes ▪ General guidance on goal setting ▪ Set initial calorie goal	Telephone (~ 20 min)
3. Getting Started with Physical Activity	▪ Safety and benefits of physical activity during pregnancy	
4. Track Down Fat	▪ Types of dietary fat ▪ Moderating total fat and saturated/trans fat intake	
5. Manage Stress	▪ Sources of stress ▪ Coping and relaxation skills	
6. Exercise Your Options	▪ Maintaining and increasing physical activity ▪ Physical activity intensity during pregnancy	
7. Celebrate Your Success	▪ Highlighting progress made to date ▪ Dietary strategies for weight management	
8. Healthy Eating Out	▪ Behavioral and dietary strategies for eating well at restaurants	
9. Handle Challenging Feelings and Triggers	▪ Problem solving challenging situations ▪ Prompts and cues; stimulus control	
10. Staying on Track During Social Activities	▪ Social support ▪ Behavioral strategies for eating in social settings	
11. Turn Setbacks into Success	▪ Relapse prevention	
12. Talk Back to Negative Thoughts	▪ Cognitive distortions ▪ Cognitive reframing	
13. Stay Motivated	▪ Highlighting progress made to date ▪ Planning for continued progress in last trimester	In-person (60 min)

The intervention promotes four goals: 1) manage GWG, 2) eat a healthful diet in appropriate portion sizes, 3) achieve 150 min per week of moderate- to vigorous-intensity physical activity, and 4) build skills to manage stress and other challenges that can get in the way of healthy lifestyle behaviors. Healthy eating recommendations emphasize vegetables, fruits, lean protein, and high fiber foods while limiting saturated and trans fats and added sugars (e.g., sugar-sweetened beverages). Physical activity recommendations emphasize being active in 10-min bouts and gradually working up to at least 30 min of activity on 5 days of the week. Recommendations encourage activities such as brisk walking and discourage activities that pose a risk for falls, loss of balance, or abdominal trauma (e.g., bicycling).

At session 1, participants will receive a body weight scale for use at home throughout pregnancy; a personalized electronic and/or paper-based graph to track their weight over time in relation to the trial goal for GWG (see Additional file 1); print materials including a guidebook of the 13 core sessions, a fat and calorie counting booklet, and paper-based self-monitoring logs; and measuring cups and spoons. Participants will first be asked to self-monitor their “baseline” dietary intake for the first week. At session 2, participants will receive an initial personalized calorie goal based on their average caloric intake in the prior week and their weight gain trajectory. For example, coaches will recommend an initial calorie goal that is equivalent to current intake for participants whose weight is at or below the target weight for their gestational age; otherwise, coaches will recommend a 200-cal reduction. At each subsequent session, coaches will engage participants in systematic problem solving to generate effective strategies to meet the calorie goal [28, 29]; and collaborate with participants to adjust the goal as needed given their weight gain trajectory. For example, coaches will recommend retaining the same calorie goal for participants who are meeting the calorie goal, are near the target weight for their gestational age, and have gained no more than 0.5 lbs. in the prior week. In contrast, coaches will recommend decreasing the goal by 200 cal for participants who are meeting the goal but have gained 0.5 lbs. or more in the past 1–2 weeks. Recommended calorie goals will never be fewer than 1200 cal at any point during the intervention. Throughout the intervention, participants will be encouraged to self-monitor their weight daily using the study-provided scale, with an emphasis on attending to patterns of weight change over time (e.g., over 1- to 2-week periods, rather than any single day’s measurement); and to self-monitor their dietary intake and physical activity daily using paper-based logs or commercially-available web- or mobile-based apps (e.g., MyFitnessPal).

Following the 13 core sessions, participants will be offered biweekly maintenance sessions by telephone until 38 weeks’ gestation. Participants and lifestyle coaches will collaboratively select topics to revisit from the core curriculum. No intervention will be provided during the postpartum period.

#### Fidelity

Our approach to intervention fidelity will follow the comprehensive framework developed by the National Institutes of Health Behavioral Change Consortium (BCC) [30, 31]. Table 2 describes strategies to address each domain of fidelity from the BCC framework: *intervention design*, *interventionist training*, *intervention delivery*, *intervention “receipt”* (participants’ understanding and ability to perform intervention skills), and *intervention “enactment”* (participants’ actual use of intervention skills). Lifestyle coaches will use a Microsoft Access tracking database to record all contacts with participants, including the duration of each call, adherence to self-monitoring, and goals set at each session. Coaches will audio record every session, with participant consent, to facilitate a systematic assessment of intervention delivery. Trained research staff will randomly select, review, and code session recordings for the delivery of key intervention content and behavior change techniques (e.g., self-monitoring, goal setting, and problem solving) using checklists developed in large part from the Behavior Change Technique Taxonomy v1 [25]. Staff will code at least 10% of the recordings for five of the core sessions (sessions 1, 2, 3, 9, and 13) which are representative of the intervention goals and behavior change techniques.

**Table 2:** Intervention fidelity plan: The GLOW trial

BCC fidelity domain	Fidelity strategies implemented in GLOW
*Intervention design*	▪ Standardize intervention dose (e.g., number and duration of sessions) ▪ Standardize interventionist credentials (registered dietician) ▪ Specify theoretical model (Behavior change techniques based on social cognitive theory and motivational interviewing)
*Interventionist training*	▪ Standardize training (e.g., training includes formal skill-building curriculum in motivational interviewing) ▪ Assess and monitor skill maintenance over time via weekly supervision, periodic re-certification and retraining, and provision of feedback
*Intervention delivery*	▪ Use an intervention protocol ▪ Document intervention delivery (e.g., number, duration, and content of sessions delivered) using an electronic tracking system and written checklists; review regularly ▪ Audiotape all sessions; code 10% of selected core sessions for key behavior change techniques, using a checklist adapted from the Behavior Change Technique Taxonomy v1 [25] ▪ Conduct periodic live observations
*Intervention receipt* and *enactment* by participants	▪ Summarize intervention content and skills at each session ▪ Document participants’ performance of intervention skills (e.g., frequency of self-monitoring) using an electronic tracking system; review regularly and provide feedback, both to participants and interventionists

BCC: National Institutes of Health Behavior Change Consortium [30, 31].

#### Intervention process measures

All participants randomized to the intervention who complete at least one of the 13 core sessions, and who do not have a pregnancy loss, will be asked to complete a mailed evaluation survey. Quantitative and qualitative survey items will assess acceptability, satisfaction, and perceived effectiveness of the intervention. Additional process measures assessed by lifestyle coaches will include attendance at each session and adherence to intervention components (e.g., frequency of weight self-monitoring).

### Data collection

Data will be collected at clinic visits conducted by trained research staff (blinded to randomization assignment) in the medical centers where women receive their usual care, and from the EHR. The baseline study clinic visit assessment will occur at approximately 10 weeks’ gestation (between 8 and 13 weeks). Follow-up study clinic visit assessments will occur at approximately 32 weeks’ gestation (30–35 weeks); delivery (within 1 week afterwards); 6 months postpartum (24–32 weeks after delivery); and 12 months postpartum (48–56 weeks after delivery). Trained research staff based at the Division of Research will oversee data quality and management.

#### Women’s weight, height, LMP, and gestational age

The date of the last menstrual period (LMP) and gestational age will be confirmed by ultrasounds performed before the baseline study clinic visit and abstracted from EHR. Pre-pregnancy weight and height, as measured by a KPNC healthcare provider within six months of the LMP, will be abstracted from the EHR. If more than one weight is recorded in the EHR, the weight closest to the LMP will be chosen. If pre-pregnancy weight is not available, we will obtain the earliest pregnancy weight measured before 10 weeks’ gestation. These measurements will be used to calculate BMI for initial assessment of eligibility. Women’s weight and height will be measured at the baseline study clinic visit at 10 weeks’ gestation by trained research staff using a standard scale and stadiometer. Women will wear light-weight clothing without shoes, with measurements taken in duplicate to the nearest 0.1 lb. and 0.1 cm, respectively. The average of the two measurements will be used if the difference between them is less than 1.0 lb. or 1.0 cm, respectively; otherwise, a third measurement will be taken. Weight will likewise be measured at study clinic visits conducted at 32 weeks’ gestation, delivery, and at 6 and 12 months postpartum. The last weight prior to delivery, measured by a KPNC healthcare provider, will be abstracted from the EHR.

#### Women’s diet and physical activity

Within the week of attending their study clinic visit at 10 weeks’ and 32 weeks’ gestation, trained staff at the Fred Hutchinson Cancer Research Center will conduct gold-standard 24-h dietary recalls on three randomly selected days over one week (including two week days and one weekend day) [32]. Mean intake over the three days will be used to estimate daily intake. Recalls will employ the Nutrition Data System for Research (NDSR), recognized as one of the best nutrient composition databases for research purposes [33–36]. The NDSR uses a computerized interface [37] to ensure uniformity of recalls conducted by interviewers trained and certified by NDSR. Women will also complete the 55-item Block Fat/Sugar/Fruit/Vegetable Screener [38] at all pregnancy and postpartum clinic visits (except delivery).

Physical activity will be assessed objectively within one week after the clinic visits at 10 and 32 weeks’ gestation. Participants will wear an ActiGraph (ActiGraph, LLC, Pensacola, FL) wGT3X-BT accelerometer on their non-dominant wrist for a 7-day period, with non-consecutive days permitted to improve adherence. Since negative feedback was received regarding overnight wear when piloting the devices, and because sleep is not an outcome of this trial, participants will be instructed to take the device off each night before bedtime and put it back on first thing in the morning. Participants will be asked not to submerge the device in water (i.e., not wear it while bathing or swimming); and to complete a written log of each time the device was put on and taken off, and the days and times they participated in activities that are not sufficiently measured by the device (e.g., swimming or biking). Participants will receive periodic text message reminders to encourage protocol adherence. Accelerometer data will be sent to the University of Tennessee, Knoxville where experts will oversee data processing using ActiLife version 6.13.3 software. Two output files will be created: a count file (i.e., exported in 1-s epochs with a low frequency extension applied) and a raw csv file (i.e., exported in 30-Hz raw acceleration values). Files will then be evaluated in R statistical software: wear time will be ascertained with the algorithm developed by Choi et al. [39–42] (count files) and metabolic equivalent (MET) values with an algorithm developed for ActiGraph wGT3X-BT devices worn on the non-dominant wrist specifically (raw files) [43, 44]; these data files will then be merged. The participant logs will be used to identify the first full calendar day of wear [42]; or, if the log is not returned, the information will be obtained via visual inspection in ActiLife. Standard days (i.e., weekday- and weekend day-specific) will be defined as the periods in which 70% of a sub-sample of participants wear the device, and a complete day defined as a day in which the device is worn for at least 80% of a standard day [45]. The objective physical activity assessments will include 4 to 7 complete days, including at least one weekend day [46]; all physical activity outcomes will be weighted (i.e., weekdays vs. weekend days) daily averages. Self-reported activity will be measured at all pregnancy and postpartum clinic visits (except delivery) using the validated Pregnancy Physical Activity Questionnaire (PPAQ) [47, 48] to assess physical activity frequency, duration, and estimated volume; and the validated Stanford Leisure-time Categorical Item (L-Cat) [49, 50] to assess relative categories of physical activity intensity and duration in relation to national recommendations.

#### Cardiometabolic markers

Women’s fasting blood samples will be collected to measure levels of glucose, insulin, adiponectin, leptin, free fatty acid, total cholesterol, triglycerides, high-density lipoprotein (HDL), low-density lipoprotein (LDL), and very low-density lipoprotein (VLDL) at 10 and 32 weeks’ gestation. Cord blood samples will be obtained to measure levels of glucose, insulin, adiponectin, leptin, free fatty acid, and C-peptide. Blood samples will be processed at the laboratory of the medical center where women will be seen for their clinic visits and delivery. Samples will then be transferred by courier under climate-controlled conditions to the Kaiser Permanente Research Bank biorepository for aliquoting, where they will be stored at − 80^°^C until being shipped to the University of Washington’s Northwest Lipid Metabolism and Diabetes Research Laboratories for analyses.

#### Infant weight, length, and percent of body fat

Infant weight and recumbent length at birth will be obtained from the EHR. In a subsample, trained staff will also measure infant recumbent length and flank skinfolds on the left side of the infant’s body at 1 to 7 days after birth, following a standard protocol.

#### Women’s body composition

In two of the largest medical centers from which women will be recruited, study staff will use an ImpediMed bioimpedance spectroscopy (BIS) device to estimate body fat mass at 10 and 32 weeks’ gestation. BIS is a non-invasive procedure that applies a very low-level electrical current to measure the body’s conductive and nonconductive tissue and fluid, including total body water (TBW). The increase in TBW accounts for the largest proportion of weight gain during pregnancy, and is highly variable [51]. The BIS device measures electrical resistance over a range of frequencies to estimate TBW as accurately as isotope dilution procedures in pregnant women [52].

#### Sociodemographic characteristics and covariates

We will assess sociodemographic and clinical characteristics including race/ethnicity, age, work and marital status, household income and size, smoking and alcohol use, and medical, weight, and reproductive history at the baseline study clinic visit at 10 weeks’ gestation. We will assess other covariates including quality of well-being (EuroQol-5D) [53], pregnancy-specific stress (Revised Prenatal Distress Questionnaire) [54], and general perceived stress (Perceived Stress Scale) [55] using validated surveys at 10 and 32 weeks’ gestation; and depression (using the Patient Health Questionnaire-8) [56] and sleep duration [57] using validated surveys at 10 and 32 weeks’ gestation and at 6 and 12 months postpartum. We will assess lactation and infant feeding [58] via interviewer-administered assessments at 6 and 12 months postpartum.

#### Potential mediators

We will assess self-efficacy [59, 60] and social support [61, 62] for healthy eating and physical activity behaviors using validated surveys at 10 and 32 weeks’ gestation. We will assess self-weighing behavior at 10 and 32 weeks’ gestation and at 6 and 12 months postpartum using a survey item adapted from prior studies [26, 27].

#### Adverse events

Perinatal adverse outcomes occurring after randomization such as pregnancy loss, gestational diabetes, preeclampsia, pregnancy-induced hypertension, Cesarean delivery, preterm birth (< 37 weeks), and low birthweight (< 2500 g), as well as possible adverse events related to physical activity such as fractures, will be abstracted from the EHR.

### Outcome measures

The primary outcome is rate of GWG per week. GWG will be defined as the difference between the last measured pregnancy weight and the pre-pregnancy weight measured within 6 months prior to the LMP (or the pregnancy weight measured before 10 weeks of gestation if pre-pregnancy weight is missing), with all weights obtained from the EHR. Rate of GWG per week will be defined as GWG divided by the number of weeks between the date of the LMP and the last measured pregnancy weight. Rate of GWG per week will also be assessed as the proportion of women exceeding the 2009 IOM GWG guidelines for rate of GWG per week. The proportion of women who exceed the IOM guidelines for appropriate weekly rate of GWG up to delivery will be defined as being above the upper limit of the first trimester recommended amount (2.0 kg) plus the upper limit of the recommended weekly rate of 0.33 kg for overweight (BMI 25.0–29.9) or 0.27 kg for obesity (BMI ≥ 30.0 kg/m^2^), times the number of gestational weeks in the 2nd and 3rd trimester.

Secondary GWG outcome measures include weekly rate of GWG between study clinic visit assessments at 10 weeks’ and 32 weeks’ gestation, total GWG, whether total GWG exceeds the 2009 IOM GWG guidelines, and the proportion of women meeting the trial goal for GWG. Weekly rate of GWG between study clinic visits at 10 weeks’ and 32 weeks’ gestation will be calculated by dividing the amount of weight gained between visits by the weeks of gestation between visits. Total GWG will be calculated by subtracting pre-pregnancy weight from the last pregnancy weight. Total GWG will be categorized in relation to the 2009 IOM guidelines for GWG specific to women’s pre-pregnancy BMI, as follows: for exceeding guidelines, GWG will be greater than 11.5 kg (overweight) or 9.0 kg (obesity); for meeting guidelines, GWG will be 7–11.5 kg (overweight) or 5–9 kg (obesity); and for below guidelines, GWG will be less than 7 kg (overweight) or 5 kg (obesity). Meeting the trial goal for GWG will be defined as total GWG that does not exceed the lower limit of the IOM-recommended range: no more than 7 kg (overweight) or 5 kg (obesity).

Other secondary outcomes include changes in women’s diet (total caloric intake and percent of calories from total fat, saturated fat, and unsaturated fat) as well as physical activity (intensity and duration), which will be assessed at 10 and 32 weeks’ gestation.

Exploratory outcomes include changes in maternal cardiometabolic markers assessed at 10 weeks and 32 weeks’ gestation, cardiometabolic profile in cord blood, postpartum weight retention at 6 and 12 months (defined as postpartum weight minus pre-pregnancy weight), birthweight for gestational age, and infant anthropometrics at 6 months and 12 months of age.

For the subsample of infants who have flank skinfold measures, infant fat mass will be calculated from birthweight, length, and flank skinfold measurements according to the equation of Catalano et al. [63], which is based on measurements of total body electrical conductivity (percent body fat is calculated as 100 x fat mass/birthweight). World Health Organization child growth standards will be used to determine weight-for-age, length-for-age, weight-for-length, and BMI-for-age percentiles [64, 65].

### Strategies to enhance clinical trial retention

To maximize retention in the trial, we will employ a multi-pronged approach focused on communication and valuing participants as essential research partners [66–68]. This includes the Methods-Motivational Interviewing (MMI) strategy of participant orientation sessions conducted prior to consent and randomization, which are designed to calibrate prospective participants’ expectations of the clinical trial process [69]. Additional strategies will include greeting cards and newsletters highlighting trial milestones, preliminary results, and participants’ contributions to the trial’s potential impact [67, 68]; communication via multiple modalities (calls, text messages, email, and mail); and reimbursement [70] with a $50 gift card for each completed study clinic visit assessment and delivery measurement, for a maximum of $250. No reimbursement will be provided for completing the intervention portion of the trial. Given the impact of staff on participants’ experiences in clinical research [71], priorities include retaining a diverse professional staff and maximizing continuity across clinic visits.

### Data safety and monitoring

The trial will be monitored by an independent Data and Safety Monitoring Board (DSMB). Goals of the DSMB will be to enhance the integrity of study procedures and data quality, provide oversight of the study’s progress, and make recommendations concerning its continuation. The DSMB will receive quarterly reports and may request additional reports due to unforeseen problems.

### Statistical analyses

Statistical analyses will be intent-to-treat, and will be conducted before and after adjusting for the variables used in the adaptive randomization procedure (age, pre-pregnancy BMI category, and race/ethnicity) [72]. We will use multiple linear regression models to examine the overall average difference between usual care and intervention conditions in mean rate of GWG and total GWG, as well as to examine the average differences in diet and physical activity variables and cardiometabolic biomarkers. We will use chi-squared tests and Poisson regression to compare conditions on the proportion of women exceeding IOM guidelines for weekly rate of GWG and total GWG, as well as to compare conditions on dichotomous outcomes.

### Power and sample size

The target sample size is 400 participants, with 200 per condition. This sample size provides 80% power to detect a between-condition difference in mean weekly rate of GWG of at least .053 kg/week, assuming 10% attrition (α = .05, two-sided test; expected standard deviation = 0.18 kg/week given preliminary data). In addition, the maximum (protective effect assumed) detectable relative risk (intervention vs. usual care) of exceeding the IOM GWG guideline is 0.75 (power = .80, α = .05, two-sided test; expected proportion in the usual care condition = 59%, given preliminary data).

## Discussion

The GLOW trial is designed to advance our knowledge about lifestyle interventions to improve weight and cardiometabolic outcomes among pregnant women with overweight or obesity and their children. GLOW is among few randomized controlled trials targeting excess GWG among diverse women in a healthcare setting, with long-term maternal and infant outcomes assessed up to 12 months after delivery. It addresses several key questions identified by the US Preventive Services Task Force in its current effort to synthesize evidence on whether lifestyle interventions effectively prevent excess GWG, postpartum weight retention, and obesity-related adverse events among women and their infants, particularly women with a high BMI before pregnancy [73].

Interventions that are feasible in the clinical settings in which women receive their routine care are urgently needed. The intervention tested in GLOW is primarily delivered by telephone; this is similar to standard-of-care telemedicine, previously shown to be effective, by which women with gestational diabetes in the KPNC health system receive weekly calls from a nurse or dietitian [74]. As such, the intervention tested here holds potential for being adopted into clinical practice, if found to be efficacious, to help pregnant women with overweight or obesity manage GWG.

Strengths of the trial design include recruitment from a racially and ethnically diverse patient population; the ability to enroll participants very early in pregnancy by leveraging the EHR; and the systematic assessment of fidelity to a theory-based intervention. Data collection strengths include the use of measured (rather than self-reported) pre-pregnancy weight; assessment of women’s objective physical activity during pregnancy; rigorous assessment of dietary intake; collection of women’s blood twice in pregnancy and cord blood to assess cardiometabolic profile; and creation of a biorepository for future research. The 12-month follow-up period will facilitate examining whether potential improvements in GWG correspond to improvements in postpartum weight retention, as has been shown in high-risk patient populations [75]. Results will be published in the peer-reviewed scientific literature.

A critical challenge in clinical trials is the need to maintain high, non-differential retention across conditions. Consistent with best practices [66], our approach will include multiple strategies, ranging from monetary reimbursement to orientation sessions conducted prior to randomization [69]. To our knowledge, GLOW is one of only two trials to implement the MMI orientation session approach among pregnant women [76].

In sum, the GLOW trial is designed to expand our understanding of the efficacy of behavioral lifestyle interventions to reduce excess gestational weight gain. This rigorous evaluation of a multi-component, theory-based intervention is designed to produce generalizable results to inform clinical practice.

## Additional file

Additional file 1: Sample gestational weight gain chart. Example of a gestational weight gain chart, illustrating a woman’s current gestational weight gain and the recommended goal, issued at session 1 for participants randomized to the GLOW lifestyle intervention. (PDF 169 kb)

## Abbreviations

BCC: Behavior Change Consortium
BIS: Bioimpedance spectroscopy
BMI: Body mass index
DPP: Diabetes Prevention Program
DSMB: Data and Safety Monitoring Board
EHR: Electronic health record
GLOW: Gestational Weight Gain and Optimal Wellness
GWG: Gestational weight gain
HDL: High-density lipoprotein
IOM: Institute of Medicine
KPNC: Kaiser Permanente Northern California
L-Cat: Stanford Leisure-time Categorical Item
LDL: Low-density lipoprotein
LMP: Last menstrual period
MET: Metabolic equivalent
MMI: Methods-Motivational Interviewing
NDSR: Nutrition Data System for Research
PPAQ: Pregnancy Physical Activity Questionnaire
SPIRIT: Standard Protocol Items: Recommendations for Interventional Trials
TBW: Total body water
VLDL: Very low-density lipoprotein
