# Supplementary figures and images for: Gestational weight gain and optimal wellness (GLOW): rationale and methods for a randomized controlled trial of a lifestyle intervention among pregnant women with overweight or obesity
Source: BMC Pregnancy Childbirth. 2019 Apr 30;19:145. doi: 10.1186/s12884-019-2293-8 (PMC6492416; doi:10.1186/s12884-019-2293-8)

# GETTING IN BALANCE

HEALTHY WEIGHT GAIN DURING PREGNANCY

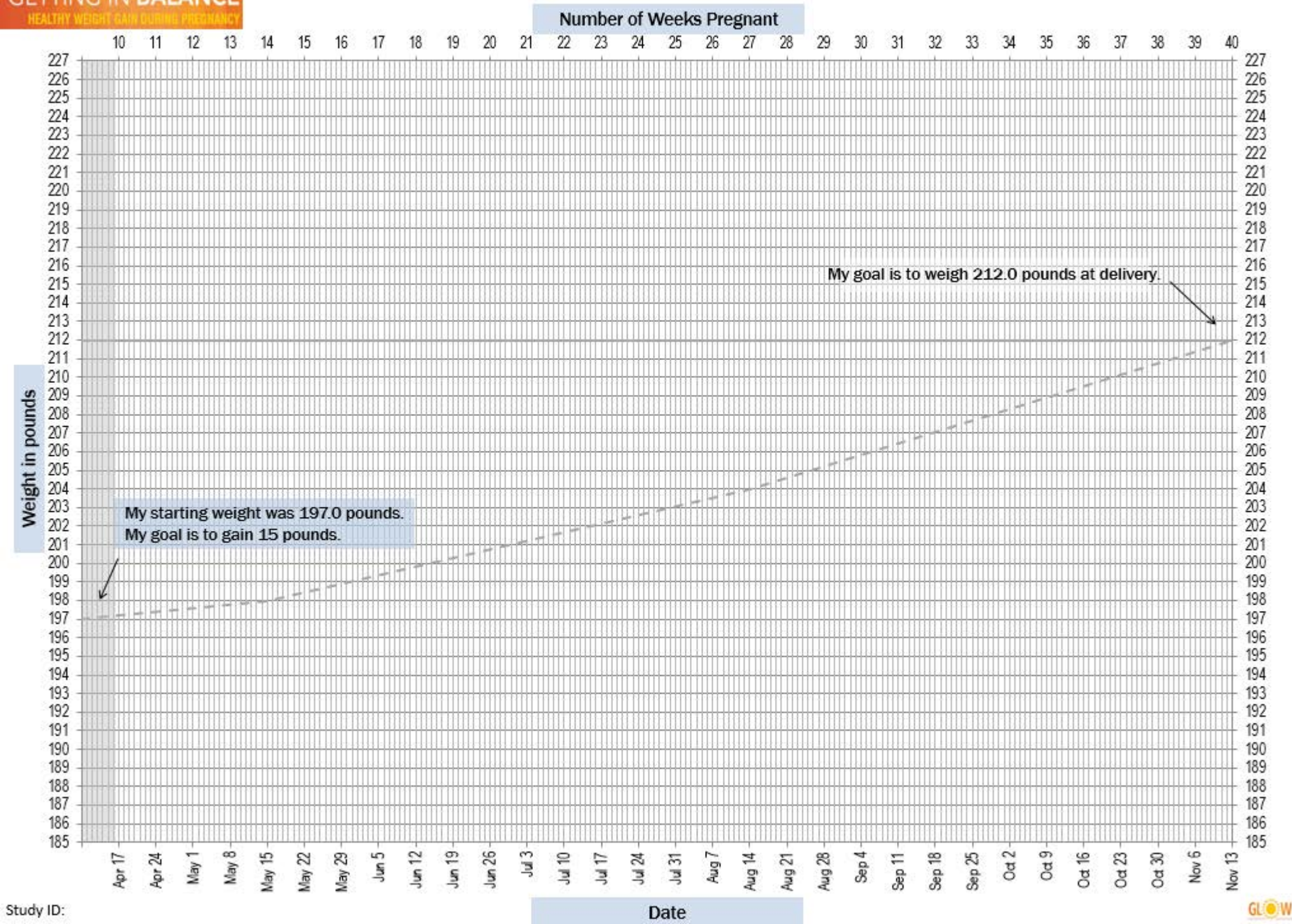

Supplement: Supplementary file 1 — Sample gestational weight gain chart. Example of a gestational weight gain chart, illustrating a woman’s current gestational weight gain and the recommended goal, issued at session 1 for participants randomized to the GLOW lifestyle intervention. (PDF 169 kb) [file 12884_2019_2293_MOESM1_ESM.pdf]
